# Fat mass and obesity-associated gene (*FTO*) is associated to eating disorders susceptibility and moderates the expression of psychopathological traits

**DOI:** 10.1371/journal.pone.0173560

**Published:** 2017-03-10

**Authors:** Giovanni Castellini, Marica Franzago, Silvia Bagnoli, Lorenzo Lelli, Michela Balsamo, Milena Mancini, Benedetta Nacmias, Valdo Ricca, Sandro Sorbi, Ivana Antonucci, Liborio Stuppia, Giovanni Stanghellini

**Affiliations:** 1 Department of Neuroscience, Psychology, Drug Research and Child Health. University of Florence, Florence, Italy; 2 Sexual Medicine and Andrology Unit, Department of Experimental, Clinical and Biomedical Sciences. University of Florence, Florence, Italy; 3 Department of Psychological, Health and Territorial Sciences, School of Medicine and Health Sciences, “G. d’Annunzio University” Chieti-Pescara, Chieti, Italy; 4 D. Portales’ University, Santiago, Chile; McMaster University, CANADA

## Abstract

Eating Disorders (EDs) show a multifactorial etiopathogenesis including environmental, psychological and biological factors. In the present study, we propose a model of interactions between genetic vulnerability—represented by Fat Mass and Obesity-Associated (*FTO*) gene—and stable psychopathological traits, such as bodily disorders and emotion dysregulation for EDs patients. The distribution of a polymorphism of the *FTO* (rs9939609 *T*>*A*) was evaluated in a series of 250 EDs patients and in a group of 119 healthy control subjects. Clinical data were collected through a face-to-face interview and several self-reported questionnaires were applied, including the Emotional Eating Scale and the IDentity and EAting disorders (IDEA) questionnaire for bodily disorders and self-identity. The *A*-allele was associated with an increased vulnerability to EDs (*AA*+*AT* genotypes frequency 72.8% in EDs vs. 52.9% in controls). The presence of the *A*-allele was associated with binge eating behavior, higher emotional eating and higher IDEA scores. Finally, the *FTO* rs9939609 SNP was found to influence the relationship between these variables, as an association between disorder of corporeality and emotional eating was found only in *A*-allele carriers. *A*-allele seems to represent a potential additive risk factor for EDs persons, with bodily disorders to develop emotional eating and binge eating behaviors.

## Introduction

The multifactorial etiopathogenesis of eating disorders (EDs) encompasses environmental, psychological and biological factors [1]. According to a gene-environmental approach, the different expression of the psychopathological core can be influenced by genetic variability, explaining a part of the variance of the susceptibility to EDs and different phenotypical features [2]. In this view, several genes have been investigated as possible candidates [3]. Among these, great interest has been recently devoted to the Fat Mass and Obesity-Associated (*FTO*) gene, previously implicated in obesity within several association studies [4,5], as a possible candidate for playing a role in the pathogenesis of EDs [6]. *FTO* encodes for an AlkB-like 2-oxoglutarate–dependent nucleic acid demethylase, a potential regulator of RNA modification, whose cellular function so far remains undefined [7].

The molecular basis of the association between the *FTO* locus and obesity have been hypothesized, even though the involved cell types and target genes remain uncharacterized [8]. Recently, several authors evidenced, with state-of-the-art chromatin-capture sequencing technologies, that noncoding variations in Single Nucleotide Polymorphisms (SNPs) within a 47-kb region in introns 1 and 2 of *FTO* do no affect the expression of this gene, but are connected, at megabase distances with the homeobox gene *IRX3* in the human, mouse and zebrafish genomes [8,9]. *IRX3* encodes a transcription factor expressed in multiple regions of the brain including cerebellum and hypothalamus, suggesting that this gene plays an important role in the regulation of body mass and composition [9].

In addition, Claussnitzer et al. [8] demonstrated that specific *FTO* variant can disrupt a conserved motif for the *ARID5B* repressor, leading to derepression of a potent preadipocyte enhancer and a doubling of *IRX3* and *IRX5* expression during early adipocyte differentiation.

Among *FTO* SNPs involved in this mechanism and associated with obesity, rs9939609 (located 82.431kb downstream of ATG start codon) is the most frequently described. The obesity predisposing risk *A*-allele is considered one of the strongest risk factor for polygenetic obesity in the Caucasian ethnic group worldwide, showing carriers of this allele with a significantly higher BMI and adiposity, as well as consuming a higher percentage of energy from fat [5, 10]. The interest in the relationship between rs9939609 and EDs pathogenesis is also due to the role of this SNP in satiety responsiveness [11] and to FTO high expression in the hypothalamic regions of the brain associated with appetite regulation [12]. Notwithstanding the hypothetically relevant connection between EDs, satiety and appetite regulation, conflicting results have so far been reported about the possible associations between *FTO* and EDs. In fact, Müller et al. [6] found an association of the *A*-allele with both diagnoses of Anorexia Nervosa (AN) and Bulimia Nervosa (BN), while Jonassaint et al. [12] did not find any significant association with AN diagnosis. However, rs9939609 was not evaluated in this latter study, which pointed at others *FTO* SNPs. Moreover, data about Binge Eating Disorder (BED) are totally lacking. Thus, these spurious results do not rule out a role of *FTO* in the susceptibility to EDs, suggesting that this association should be further investigated using a more refined assessment of ED psychopathology. Also, phenotypes defined by DSM diagnoses such as AN, BN, BED are known to be unstable through time [13, 14, 15] and unsuitable for correlations with genetic polymorphisms.

Our hypothesis is that significant correlations can be found between *FTO* and ED phenotypes associated with stable traits such as disorders of corporeality. Indeed, they have been found to often precede eating disorders [16] and to predict long term outcome [14, 17]. In this view, in the present study we propose a model of interactions between genetic vulnerability represented by a specific polymorphism and stable psychopathological traits, resulting in specific pathological eating attitudes and behavior, which can be considered as endophenotypes (intermediate phenotypes) of this condition. In particular, high-risk variant in *FTO* could affect eating behaviours (i.e. by means of binge-eating episodes) and promote excessive weight gain and obesity.

The main aim of the present study is to investigate the role of *A*-allele at rs9939609 as a risk factor for Eating Disorder and in particular for the above mentioned specific ED psychopathology aspects.

## Materials and methods

The distribution of the *FTO* rs9939609 (*T*>*A*) was evaluated in a series of 250 Italian, Caucasian, EDs patients referred to the outpatient clinic for EDs at the Florence University School of Medicine, and in a group of 119 Caucasian healthy sex matched control subjects. All diagnostic procedures (apart from the extraction of blood samples and genetic analyses) and psychometric tests are part of the routine clinical assessment for EDs patients regularly performed at our clinic, as previously described [18]. Study procedures were fully explained during the first routine visit and prior to the collection of data; after which the patients were asked to provide two different written informed consents, one for participation in the clinical study and one for the genetic analysis. The study protocol was performed in compliance with the Declaration of Helsinki and was approved by the Ethics Committee of G. d’Annunzio University and Careggi Hospital University, respectively. The detailed study design has already been described in two earlier studies reported by our group [18, 19].

### Participants

EDs patients attending the outpatient clinic for EDs of Florence, Italy, between July 2010 and March 2014 were enrolled in the study, provided they met the following inclusion criteria: aged between 18 and 60 years; current DSM-IV diagnosis of AN and BN assessed by means of the Structured Clinical Interview for DSM-IV [20]. The study design and methodology was adopted from our previous studies [18]. The diagnoses were based on the current symptomatology at referral. Patients were included if they reported at least 3 years of a stable diagnosis, according to the Diagnostic and Statistical Manual of Mental Disorder Fourth Edition (DSM-IV) criteria [21]. The Structured Clinical interview for DSM-IV was also used to identify the presence of comorbid axis I mental disorders. Exclusion criteria were as follows: a BMI of <14 kg/m2, comorbid schizophrenia, bipolar I disorder, illiteracy, intellectual disability; severe medical conditions; current use of psychoactive medications, with the exception of antidepressant medication and benzodiazepines.

Of the 273 subjects who met the participation criteria, 250 agreed to participate in the study. In particular, 134 patients were affected by AN [56 with AN-B/P subtype (BP), 78 with AN-restricting subtype (AN-R)] (mean age ± SD = 29.8 ± 10.3 years) and 116 by BN (mean age ± SD = 30.7 ± 10.8 years). Patients were compared with a group of 119 Caucasian unrelated controls (mean age ± SD = 24.4 ± 4.2 years), consecutively recruited through local advertisements at the “G. d’Annunzio” University of Chieti-Pescara. The controls were carefully interviewed in order to exclude any history of an ED, any actual psychiatric axis I disorder or the presence of a first-degree relative suffering from an ED.

### Study design

The study was based on a naturalistic approach. The clinical assessment, as well as the collection of blood samples, was conducted on the first day of admission.

### Assessment

The clinical assessment is described more in depth in previous publications from our group [18, 19]. Socio-demographic, psychopathological, and clinical data were collected through a face-to-face interview by two expert psychiatrists (G.C., V.R.). The ED diagnoses were performed using the structured clinical interview for DSM-IV axis I disorders [20]. Eating attitudes and behaviors were specifically investigated by means of the Eating Disorder Examination Questionnaire (EDE-Q) [22]. Emotional eating defined as “tendency to eating in response to different emotions” was assessed by means of the Emotional Eating Scale (EES) [23].

For further characterization of the psychopathological features of the patients, the Beck Depression Inventory (BDI), a widely used and well established measure of current depression level and symptoms [24], the Spielberg’s State-Trait Anxiety Inventory (STAI), to measure levels of trait anxiety [25] and the IDentity and EAting disorders (IDEA) questionnaire [26] were administered.

IDEA represents a multidimensional, brief, versatile, easy-to-perform self-reported instrument for clinical evaluation, assessing bodily disorders, and of personal identity, specifically associated with the core features of ED psychopathology. The questionnaire consists of 23 items evaluated by a likert scale from (0) “not at all” to (4) “very much”.

The questionnaire provided a total score and four subscales: ‘Feeling oneself only through the gaze of the other and defining oneself only through the evaluation of the other’ (GEO), ‘feeling oneself only through objective measures’ (OM), feeling extraneous from one’s own body’ (EB), and ‘feeling oneself only through starvation’ (S) [26]. Total IDEA scores were calculated by averaging participants’ rating across the 23 items. The test showed good test-retest reliability, and internal consistency, both in clinical [26] and general population [27].

The total score of the BDI (ranges from 0 to 63) and STAI (ranges from 20 to 80) were calculated by summing up item scores. Instead, EDE-Q and EES total scores were calculated by averaging participants’ rating, respectively.

### Genetic analysis

Genomic DNA was isolated from peripheral blood lymphocytes of ED patients and buccal swabs of controls using standard methods. All DNA samples were amplified by Polymerase Chain Reaction (PCR) performed in 25 μl reaction volume containing 50 ng of genomic DNA in a AB Applied Biosystem 2720 thermal cycler (Applied System, Foster City, CA), using the KAPA Taq DNA polymerase (Resnova, Genzano, Italy). The amplification products was submitted to: i) direct sequencing procedure using BigDye Term v3.1 CycleSeq Kit (Life Technologies, Monza, Italy) followed by automatic sequencing analysis (ABI PRISM 3130XL) or ii) High Resolution Melting (HRM) using PikoReal™ Real-Time PCR System (Thermo Scientific) according to the manufacturer’s instructions.

### Statistical analysis

#### Clinical variables

Continuous variables were reported as mean ± SD, whereas categorical variables were reported as numbers and percentages. The independent sample t test and the χ^2^ were used for continuous and categorical variables respectively, to compare AN and BN groups.

#### Genotype analyses

A principle component analysis (PCA), with Varimax rotation, using genotype as a qualitative variable was performed. PCA applied to genotype data can be used to calculate principal components (PCs) that explain differences among the sample individuals in the genetic data. Based on the presence of each genotype (*AA*, *AT*, and *TT*) for each subject of the overall sample, the *FTO* SNP was transformed and coded into three binary variables (0 = absence of genotype on each subject; 1 = presence of genotype on each subject) named: *AA*, *AT* and *TT* variables.

According to Kaiser’s criterion [28], PCs with eigenvalues greater than 1 were extracted. Component Plot in rotated space was also provided to a better visual representation of the loadings plotted in a 2-dimensional space. Differences on each of the genotype components indicated by the PCA between patients and healthy control subjects, as well as among AN and BN subgroups and healthy control subjects, were tested using one-way analysis of variance. In addition, correlation analysis among the genotype components indicated by the PCA, IDEA and EES scores, and some socio-demographic variables (age and BMI) was performed.

First of all, genetic analyses were performed considering AN and BN as separate groups, as they may show different genetic components. Considering the well demonstrated instability of DSM diagnostic categories, we further evaluated genotypes on the basis of behavioral and psychopathological features.

The overall association between the ED (AN or BN) and the *FTO* genotypes (*AA*, *AT*, *TT*), was tested. Considering the heterogeneity of the adopted models for this SNP in the scientific literature, genotype analyses were performed assuming dominant (*TT* vs. *TA*+*AA)*, co-dominant (*TT* vs. *AT* vs. *AA*) and recessive (*TT*+*TA* vs. *AA*) models by means of the χ2 statistics.

We compared genotype distribution separately between ED and healthy controls. Independent sample t test was performed to evaluate BMI, EDE-Q, total and subscale scores, EES, BDI, and STAI, IDEA total and subscales scores differences in the patients with different *FTO* genotypes. General Linear Models were adopted to test the moderating effects of *FTO* genotypes on the association between IDEA scores and psychopathology. In particular, a first model was tested with Emotional Eating total score as dependent variable (EDs psychopathology) and age, BMI and IDEA scores as covariates. Then, in a second model, we entered IDEA by FTO genotypes *(AA*, *AT*, *TT*) interaction. The Statistical Package for the Social Sciences for Windows SPSS (IBM, 2011) version 20.0 was used for data analysis.

## Results

The demographic and clinical characteristics of ED and control groups are reported in Table 1. As expected, AN patients showed a lower BMI as compared with controls as well as with BN patients. Patients and controls did not differ in terms of education (mean years of education 12.5 ± 1.7 vs 12.8 ± 1.4; t = 1.7, p = 0.08). Other comparisons between clinical groups were not significant.

**Table 1:** General, Psychopathological and Clinical Characteristics of the Eating Disorders groups.

	AN (n: 134)	BN (n: 116)	Χ^2^; Independent sample t test	Healthy controls (n: 119)
Age (years)	29.8 ± 10.3	30.7 ± 10.8	0.37	24.4 ± 4.2^§§, ##^
SEX (women)	126 (94.0%)	114 (98.3%)	2.91	119
BMI (kg/m^2^)	16.1 ± 1.5	21.2 ± 2.6	19.4 **	22.2 ± 3.1^§§^
BDI	21 ± 11	21 ± 9	-0.19	-
STAI-S	39 ± 22	38 ± 24	0.15	-
STAI-T	45 ± 26	40 ± 26	0.82	-
EDE-Q total score	3.1 ± 1.3	3.0 ± 1.4	0.37	-
EDE-Q restraint	2.7 ± 1.5	2.7 ± 1.8	-0.31	-
EDE-Q eating concern	2.8 ± 1.4	2.6 ± 1.6	0.79	-
EDE-Q weight concern	3.3 ± 1.5	3.2 ± 1.7	0.35	-
EDE-Q shape concern	3.8 ± 1.5	3.6 ± 1.7	0.73	-
EES total score	1.6 ± 1.0	1.7 ± 1.1	-0.56	-
IDEA total score	1.5 ± 0.9	1.4 ± 0.9	1.31	0.8 ± 0.7^§§, ##^
IDEA GEO	1.5 ± 1.0	1.3 ± 1.1	1.67	0.8 ± 0.8^§§, ##^
IDEA OM	1.9 ± 1.2	1.8 ± 1.1	0.54	0.9 ± 1.0^§§, ##^
IDEA EB	1.5 ± 1.1	1.3 ± 1.1	1.05	0.5 ± 0.7^§§, ##^
IDEA S	1.3 ± 1.1	1.3 ± 1.1	0.04	1.2 ±0.9^§§, ##^

Statistics: Data are reported as numbers (percentage), and mean ± SD.

Abbreviations: AN, anorexia nervosa; BN, bulimia nervosa; BMI, body mass index; BDI, beck depression inventory; STAI, state/trait anxiety inventory; EDE-Q, Eating Disorder Examination Questionnaire; EES: Emotional Eating Scale; IDEA: Identity and Eating Disorders Questionnaires; GEO: ‘feeling oneself only through the gaze of the other and defining oneself only through the evaluation of the other’; OM: ‘feeling oneself only through objective measures’; EB: ‘feeling extraneous from one’s own body’; S: ‘feeling oneself only through starvation’.

Comparisons between AN and BN patients—**P <0.01. Comparisons between healthy controls and AN patients - §§: p<0.01. Comparisons between healthy controls and BN patients—##: p<0.01.

**Fig 1:**
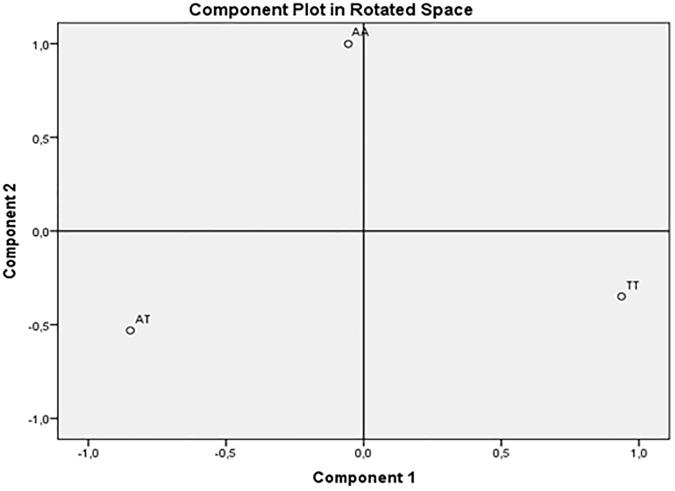
Component Plot in Rotated Space.

By applying PCA, two orthogonal (not correlated) PC, with eigenvalues greater than 1, best explained our genotypes sample structure. The rotated component matrix showed that the first PC was composed by *TT* genotype variable, which accounted for 54% of the total variance (eigenvalue = 1.62). The second PC was identified by *AA* genotype variable, explaining 46% of the total variance (eigenvalue = 1.38). Differently, the *AT* genotype variable showed double salient loadings on both the components, thus making it not interpretable [29]. Component Plot in rotated space showed how closely related the variables were each other and to the two components. As you can see in Fig 1, the two genotype variables were orthogonal: *TT* genotype variable showed factor loadings close to 1 on the first component, and close to 0 on the second, while *AA* genotype variable showed loadings close to 0 on the first component, and near 1 on the second. AT genotype variable had negative and moderate loadings on both the components, thus it resulted to be not easily identifiable in the factor space. Thus, the factor solution that met best the criterion of simple structure as defined by Thurstone [30, 31], in order to guarantee easy interpretability of the extracted components and good replicability of the results, included two components: *AA* and *TT* genotype components.

Significant difference (*F*_*1*, *367*_ = 8.28, *p* < .01) on *AA* and *TT* genotype components between patients and healthy control subjects was found.

Healthy control subjects (M_TT_ = |.215| M_AA_ = |.237| SD_TT =_ 1.079_,_ SD_AA_ = .796) reported significantly higher means on AA and TT genotype components, compared to patients (M_AA_ = |.102|, M_TT_ = |.113|; SD_TT_ = .945, SD_AA_ = 1.066). Also, significant differences on *TT* (*F*_*2*, *368*_ = 4,21, *p* < .05) and *AA* (*F*_*2*, *368*_ = 9,99, *p* < .001) genotype components between AN subgroup, BN subgroup and healthy control subjects were found. Specifically, on *TT* genotype component, the Bonferroni post-hoc revealed that healthy control subjects showed significantly higher mean (M = |.21|, SD = |1.07|) compared to AN subgroup (M = |.12|, SD = |.98|), while no significant differences between BN subgroup mean (M = |.07|, SD = |.89|) versus AN group mean and versus healthy control subjects were found. As regards *AA* genotype component, BN subgroup showed significantly higher mean (M = |.32|, SD = |1.14|) compared to AN subgroup (M = |.06|, SD = |.97|) and healthy control subjects (M = |.24|, SD = |.80|).

Summing up, the PCA best explained differences among the sample individuals in the genotype data.

Also, correlations of the two genotype components with IDEA and EES scores, as well as with socio-demographic variables (age and BMI), were tested. Results showed that *TT* genotype component correlated significantly with IDEA EB (*r* = -.03, *p*>.01), while *AA* genotype component correlated significantly with IDEA GEO (*r* = .12, *p*>.05), IDEA EB (*r* = .11, *p*>.05), and EES total score (*r* = .12, *p*>.05).

The distribution of genotypes of rs9939609 in controls and EDs patients as a whole sample and according to different categories (AN, BN) are reported in Table 2. A significant difference was detected in terms of genotype distribution between healthy controls and ED patients, being *A*-allele carriers (*AA* and *AT* genotypes) significantly more frequent in EDs patients than in healthy controls (72.8% vs 52.9%, Χ^2^ = 14.2; p<0.001).

**Table 2:** Distribution of rs9939609 genotypes according to diagnoses, along with relative odds ratios.

GENETIC MODEL								
		CO-DOMINANT MODEL			DOMINANT MODEL		RECESSIVE MODEL	
Groups	No.	Genotype n (%)			Genotype n (%)		Genotype n (%)	
		AA	AT	TT	AA +AT (coded as 1)	TT (coded as 0)	AA (coded as 1)	AT + TT (coded as 0)
Healthy controls	119	16 (13.4%)	47 (39.5%)	56 (47.1%)	63 (52.9%)	56 (47.1%)	16 (13.4%)	103 (86,6%)
EDs	250	73 (29.2%)	109 (43.6%)	68 (27.2%)	182 (72.8%)	68 (27.2%)	73 (29.2%)	177 (70,8%)
**OR (CI) *P***		χ^2^ = 18.08; p<0.001			χ^2^ = 14.2; p<0.001; OR 95% CI = 2.37; 1.50–3.75		χ^2^ = 10.9; p = 0.001;OR 95% CI = 2.65; 1.46–4.80	
BN	116	43 (37.1%)	41 (35.3%)	32 (27.6%)	84 (72.4%)	32 (27.6%)	43 (37.1%)	73 (62,9%)
AN	134	30 (22.4%)	68 (50.7%)	36 (26.9%)	98 (73.1%)	36 (26.9%)	30 (22.4%)	104 (77,6%)
**OR (CI) *P***		Ns			χ^2^ = 0.02; p = 0.98; OR 95% CI = 1.03; 0.59–1.81		χ^2^ = 6.48; p = 0.01; OR 95% CI = 2.04; 1.17–3.55	
Binge eating patients	172	58 (33.7%)	74 (43.0%)	40 (23.3%)	132 (76.7%)	40 (23.3%)	58 (33.7%)	114 (66,3%)
AN restricter	78	15 (19.2%)	35 (44.9%)	28 (35.9%)	50 (64.1%)	28 (35.9%)	15 (19.2%)	63 (80,8%)
**OR (CI) *P***		χ^2^ = 7.05; p = 0.02			χ^2^ = 4.33; p = 0.037; OR 95% CI = 1.84; 1.03–3.30		χ^2^ = 5.45; p = 0.020; OR 95% CI = 2.13; 1.12–4.07	

Legend: Binge eating patients include Bulimia Nervosa and Anorexia Nervosa binge/purge patients. ns: Not statistical significant.

Abbreviations: EDs: Eating Disorders; AN: Anorexia Nervosa; BN: Bulimia Nervosa.

Odds ratio with 95% CIs were used for dominant model (*AA* + *AT* vs. *TT*) in ED subjects versus control subjects (OR: 2.37; 95% CI: 1.50–3.75; *P* = 0.0002).

On the other hand, recessive model showed that *AA* genotype allele (*AT* + *TT* vs *AA*) was more frequent in patients as compared with healthy controls (χ2 = 10.9; p = 0.001; OR 95% CI = 2.65; 1. 46–4.80).

Within the ED patient group, no significant differences in the frequency of the *A*-allele were detected between AN and BN (72.4% vs. 73.1%), while recessive model showed a significant positive association of *AA* genotype with BN (χ2 = 6.48; p = 0.011; OR 95% CI = 2.0; 1.17–3.55).

A modest association was found between *A*-allele and binge eating behaviors (BN and AN binge/purge patients), being *A*-allele carriers more frequent among these patients as compared to ANR patients (76.7% vs. 64.1%, Χ^2^ = 4.31; p = 0.037). A modest association was found in recessive model between *AA* genotype and binge eating behaviors (χ2 = 5.45; p = 0.02; OR 95% CI = 2.13; 1.12–4.07).

In the controls, the *FTO* rs9939609 genotypic frequency did not deviate from Hardy-Weinberg equilibrium (HWE) (p = 0.23), whereas a marginal deviation was found in EDs (p = 0.04).

The comparison of clinical variables, in relation to rs9939609 polymorphism, are reported in Table 3. *A*-allele carriers, as compared to *TT* genotype carriers, reported significantly higher emotional eating scores (1.7±1.0 vs. 1.3±1.0, χ^2^ = -2.65; p<0.01) and IDEA subscale scores, in particular: ‘feeling oneself only through objective measures’ (2.1 ± 1.0 vs. 1.7 ± 1.2, χ^2^ = -1.96; p<0.05) and ‘feeling extraneous from one’s own body’ (1.7 ± 1.2 vs. 1.2 ± 1.0, χ^2^ = -1.29; p<0.05).

**Table 3:** Comparison of clinical variables according to FTO genotypes.

	A-allele carriers (AA and AT genotypes; n: 182)	TT genotype (n: 68)	Χ^2^; Independent sample t test
SEX (women)	182 (97.1%)	68 (95.6%)	0.27
BMI (kg/m^2^)	19.0 ± 4.0	18.7 ± 3.3	0.51
BDI	21 ± 11	23 ± 7	0.77
STAI-S	38 ± 24	38 ± 22	- 0.01
STAI-T	42 ± 26	43 ± 25	0.09
EDE-Q total score	3.1 ± 1.4	2.8 ± 1.2	- 1.21
EDE-Q restraint	2.7 ± 1.7	2.7 ± 1.3	0.02
EDE-Q eating concern	2.7 ± 1.5	2.5 ± 1.4	- 0.83
EDE-Q weight concern	3.3 ± 1.7	2.9 ± 1.4	- 1.38
EDE-Q shape concern	3.7 ± 1.7	3.4 ± 1.7	- 1.03
EES total score	1.7 ± 1.0	1.3 ± 1.0	- 2.65**
IDEA total score	1.4 ± 0.9	1.5 ± 0.8	0.49
IDEA GEO	1.4 ± 1.1	1.4 ± 1.0	0.11
IDEA OM	2.1 ± 1.0	1.7 ± 1.2	-1.96*
IDEA EB	1.7 ± 1.2	1.2 ± 1.0	-1.29*
IDEA S	1.3 ± 1.1	1.3 ± 1.0	-0.15

Statistics: Data are reported as numbers (percentage), and mean ± SD.

Abbreviations: AN, anorexia nervosa; BN, bulimia nervosa; BMI, body mass index; BDI, beck depression inventory; STAI, State/Trait anxiety inventory; EDE-Q, Eating Disorder Examination Questionnaire; EES: Emotional Eating Scale; IDEA: Identity and Eating Disorders Questionnaires; GEO: ‘feeling oneself only through the gaze of the other and defining oneself only through the evaluation of the other’; OM: ‘feeling oneself only through objective measures’; EB: ‘feeling extraneous from one’s own body’; S: ‘feeling oneself only through starvation’.

*P <0.05.

**P <0.01.

**Fig 2:**
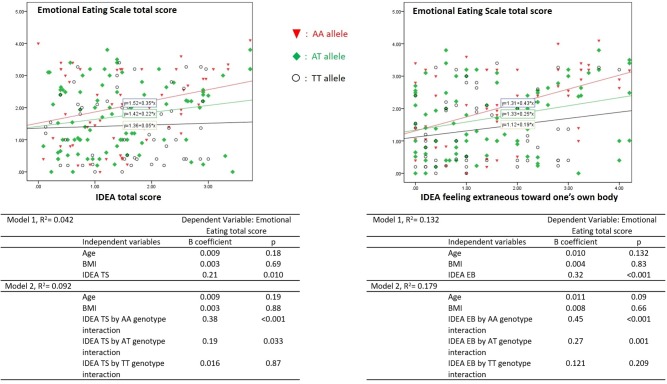
Relationship between IDEA scores and Emotional Eating in Eating Disorders patients (n: 250): moderating effect of *FTO* genotypes. **Statistics:** Tables report General Linear Models *FTO* by IDEA interaction. The first models tested the effects of IDEA scores on Emotional Eating, entering age, and BMI. In the second step *FTO* genotype interaction was added. Tables showed the increasing effect of different FTO genotypes (*AA*, *AT*, *TT*).

Finally, General Linear Model showed that *FTO* genotypes significantly moderated the association between emotional eating and IDEA scores (Fig 2). A significant effect was added entering *FTO* genotype in the model of association between Emotional Eating total score and IDEA TS (R squared from = 0.04 to 0.09), and IDEA EB (R squared from = 0.13 to 0.18). In particular, the models showed an increasing effect for the interaction according to different genotypes (IDEA TS, *AA*: p<0.001, *AT*: p = 0.03, *TT*: p = 0.87; IDEA EB: *AA*: p<0.001, *AT*: p = 0.001, TT: p = 0.21).

## Discussion

In the present study, we evaluated the *FTO* rs9939609 variant as a risk factor for EDs, because of its well-known role in controlling energy homeostasis and eating behavior [32]. To the best of our knowledge, this is one of the very few studies considering the possible role of *FTO* in the different expression of EDs specific psychopathology [6, 12]. In general, our preliminary study showed that

FTO rs9939609 variant represents a putative risk factor for people with high disorder of bodily disorders to develop emotional eating. As extensively reported in the literature, emotional eating is a pathological behavior, which accounts for development of obesity and for several pathological eating behaviors such as binge eating [33, 34].

As a first result, in both dominant and recessive models, we found a significant association of rs9939609 *A*-allele with vulnerability to EDs, being this allele more frequent among EDs patients than in controls. In particular, this association was related to the binge eating behavior, present in both AN B/P and BN patients, rather than to the DSM diagnostic category.

Secondly, the *A*-allele was associated with an increased severity of specific EDs psychopathological features, including emotional eating and disorder of corporeality, and it shows a moderating effect between these variables. Many studies have analysed the *FTO* variants role on metabolism, but only few studies have explored the association between *FTO* variants and cerebral activity. Two groups explored how *FTO*’s putative demethylase action affect complex human phenotypes [35, 36]. Karra et al. [36], using MRI techniques, demonstrated that *FTO* genotype modulates the neural responses to food cues and modifies the responsivity to ghrelin levels in the fasted state in normal-weight volunteers. These findings provide a possible explanation to the link between the *A*-risk allele and obesity predisposition through the regulation of the reward and appetite regions. Hess et al. [35] demonstrated the tissue- or even cell-type-specific role of *FTO* by regulating mRNA subsets relevant to the physiological function of the tissue. This study described a potential role of FTO to influence food intake by altering D2 receptor-dependent feeding behaviour [35].

In a behavioral and fMRI study, Sevgi et al. [37] demonstrated that variants of the *FTO* gene affect dopamine (D2)-dependent mesocorticolimbic-prefrontal responses that may influence reward learning and behavioral responses associated with learning from negative outcome in humans. In addition, Olivo et al. [38] studied the interaction between genetics, neural patterns, and behavioral measures in determining the obesity risk. The authors suggested that the *AA* carriers show neural connectivity patterns that might more closely influence the sensitivity toward punishment and reward. In turn, social and environmental factors acting on this neural substrate might increase the susceptibility to obesity.

Moreover, a recent study demonstrated how *AA* and *TT* genotype are associated with differential neural processing of food images of different caloric content: in fact, the *AA* genotype had increased brain activity specifically in areas important for emotion (cingulate gyrus), memory, and self-image (cuneus and precuneus) and reward (putamen) compared with the *TT* genotype [39].

Different mechanisms for explaining *FTO*’s role in the association with ED susceptibility can be proposed. A possible role in energy homeostasis is supported by studies performed on humans, mice and rodents that have suggested that *FTO* non coding variants could affect hypothalamic mRNA expression, in turn able to regulate food intake, circulating glucose levels, weight status and energy expenditure [7, 40].

Despite all these emerging data, a study by Smemo et al. [9] pursued functional interactions between obesity-linked SNPs and neighbouring genes. In fact, the authors demonstrated, for the first time, an association between intronic variance within *FTO* and IRX3 expression, suggesting a broader role for *FTO* beyond that of regulating fat mass [9]. The promoter of *IRX3* directly binds to an enhancer sequence within the first *FTO* intron. The *IRX3* role in the regulation of body mass and composition is shown by a reduction in body weight of 25 to 30% in Irx3-deficient mice, through increase in basal metabolic rate with browning of white adipose tissue. In addition, hypothalamus-specific dominant-negative form of Irx3 mice reproduces the metabolic phenotype of Irx3-deficient mice [9].

Therefore, *FTO* variants may represent a risk factor for overweight condition, in turn considered a frequent antecedent of both AN and BN patients [22]. Accordingly, previous studies indicated an association of the rs9939609 risk *A*-allele with increased energy intake and decreased satiety [11]. According to Müller et al., [6], it can be suggested that the observed association of *FTO* with EDs might be independent from the elevating effect of this variant on BMI, and that both a weight-elevating effect of the risk *A*-allele and a modulation of eating behavior and satiety accounts for the observed associations.

The association of the *A*-allele at rs9939609 with AN was already observed by Muller et al. [6]. Furthermore, Boraska et al. [41] performed a genome-wide association scan (GWAS) of anorexia nervosa. When the authors compared 76 variants (53 independent) from the AN results with 89 established BMI/obesity SNPs, *FTO* variant and other 4 SNPs (inside other genes) had P<0.05 (binomial P = 0.1906). Twenty-six of the 53 SNPs had the same direction of effect in AN and BMI/obesity (binomial P = 1) and 3 variants included *FTO* had P<0.05 (binomial P value = 0.0084), indicating modest enrichment of nominally associated SNP from extreme obesity in their discovery dataset [41]. More recently, through cross-trait analysis of the 1000 SNPs and functional ex vivo studies, Hinney et al [42] identified three genetic loci involved in both anorexia nervosa risk and variation of BMI. None of the nine SNPs located in these loci have previously been associated to either AN or BMI/obesity or other psychiatric disorders. However, no SNPs in *FTO* gene reached genome-wide significance in data.

Of particular interest is the association between the *A*-allele and the endophenotypes of EDs. Interest in endophenotypes has been stimulated by the difficulties encountered in establishing specific genetic causes for complex disorders by classical linkage or association designs. In this context, the concept of endophenotype may be of relevant value in order to bridge the gap between the genotype and the phenotype, through the deconstruction of the clinical phenotype into variables hypothetically more proximal to genetic effects.

In this view, we attempted to overcome the behavioral level of the EDs psychopathology, characterized by diagnostic instability [14, 43], by considering more stable quantitative traits, such as emotional eating and disorders of corporeality, as candidate phenotypes relevant to EDs pathology [44, 45].

Emotional Eating (EE)–defined as tendency to eat in response to a range of negative emotions such as anxiety, depression, anger and loneliness, to cope with negative affect—has been identified as a possible factor triggering binge eating in BN [23, 46] and binge eating disorder [47]. A second candidate endophenotype for EDs investigated in this study was represented by the bodily disorders [26, 27, 48]. Pathological concerns about body shape and weight are considered as secondary epiphenomena to a more profound pathological core [17, 43]. ED patients complain about their being alienated from their own body and emotions, and experience body as an object that is looked at and judged by other subjects [26]. Especially, feeling extraneous from one’s own body may feature as a possible factor for abnormal eating patterns via emotional dysregulation and/or the dysregulation of bodily-mediated satiety responsiveness. According to this model, the cenesthetic apprehension of one’s own body is the more primitive and basic form of self-awareness, and patients with EDs often report–with different extents of insight–their difficulties in perceiving their emotions and that they do not “feel” themselves [27]. In this study, we found a significant association between emotional eating and the IDEA subscale measuring feeling extraneous from one’s own body. Moreover, we found that the *A*-allele of the rs9939609 represented a moderator of the association between emotional eating and disorder of corporeality and identity measured by IDEA. Therefore, our findings suggest bodily disorders in the individuals with the A-allele, is more likely to favor emotional eating as well as binge eating phenomena.

Overall, the results confirm the importance of a fine-tuned characterization of the EDs psychopathology based on qualitative and subjective rather than quantitative measures.

However, the present study has the limitation of the sample pool size being relatively small. Future studies with a larger sample size are required. It should be noted that due to our small sample size, we possibly overestimated the odds ratio of the minor allele.

Finally, the information regarding the population of patients seeking treatment cannot be generalized to all people presenting pathological eating behaviors. It is possible that some aspects of the described clinical features are state-dependent. However, it is important to note that recent observations in clinical [14, 17] and general population [27] confirmed that disorders of corporeality related with EDs are more stable across time as compared to DSM diagnoses, which are typically prone to the so called diagnostic crossover [13]. Indeed, the clear association of *FTO* genotypes with measures of disorders of corporeality rather than with DSM diagnoses confirmed the hypothesis that they can represent stable trait of EDs. In addition, the SNP diverts from equilibrium of these subjects: this would be consistent with the association results of binge eating traits.

Furthermore, it should be remembered that the patients were included if they reported at least 3 years of a stable diagnosis. This may explain in part the lack of age matching in between cases and controls (there were significantly younger in the control group).
